# Supplementary material for: Structural basis for a nucleoporin exportin complex between RanBP2, SUMO1-RanGAP1, the E2 Ubc9, Crm1 and the Ran GTPase
Source: Nat Commun. 2025 Jul 11;16:6403. doi: 10.1038/s41467-025-61694-1 (PMC12246119; doi:10.1038/s41467-025-61694-1)
Supplement: Supplementary file 1 — Supplementary Information [file 41467_2025_61694_MOESM1_ESM.pdf]

Supplementary Data Figures

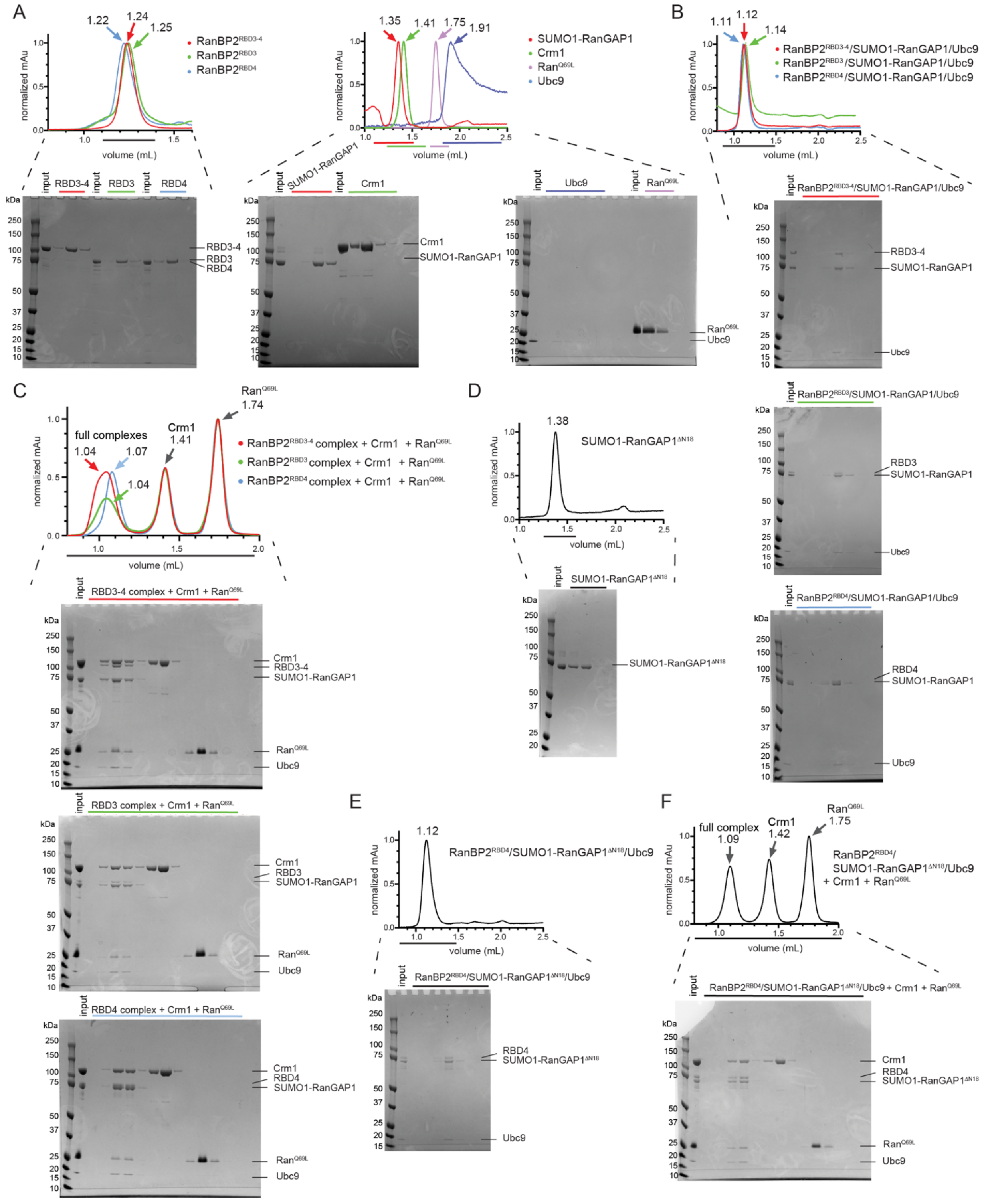

Supplementary Figure 1. Size exclusion chromatography and SDS-PAGE analyses of RanBP2 complexes, subcomplexes, and individual components

A RanBP2 variants (2 μM), SUMO1-RanGAP1 (3 μM), Crm1 (6 μM), Ran^{Q69L}(GTP) (20 μM), or Ubc9 (3.5 μM) were individually injected onto a Superdex Increase 3.2/300 column. Elution volumes are indicated above peaks and fractions collected for SDS-PAGE analysis are indicated below. mAu values were normalized against the maximum mAu value in the corresponding chromatograph.

B Reconstituted and purified (see Methods for details) RanBP2/SUMO1-RanGAP1/Ubc9 subcomplexes (1 μM) containing either $RanBP2^{RBD3-4}$, $RanBP2^{RBD3}$, or $RanBP2^{RBD4}$ were injected onto the Superdex Increase 3.2/300 as in (A). Labels and normalization as in (A).

C Full chromatographs and respective SDS-PAGE gels for SEC runs shown in Fig. 1C. Reconstituted RanBP2/SUMO1-RanGAP1/Ubc9 subcomplexes (2 μM) containing either $RanBP2^{RBD3-4}$, $RanBP2^{RBD3}$, or $RanBP2^{RBD4}$ were combined with Crm1 (6 μM) and Ran^{Q69L}(GTP) (20 μM) and injected onto Superdex Increase 3.2/300 as in (A) and (B). Labels and normalization as in (A) and (B).

D-F Analysis of SUMO1-$RanGAP1^{\Delta N18}$ (3 μM) (D), reconstituted $RanBP2^{RBD4}$/SUMO1-$RanGAP1^{\Delta N18}$/Ubc9 subcomplex (1 μM) (E), or reconstituted $RanBP2^{RBD4}$/SUMO1-$RanGAP1^{\Delta N18}$/Ubc9 (2 μM) combined with Crm1 (6 μM) and Ran^{Q69L}(GTP) (20 μM) (F) via size exclusion chromatography on the Superdex Increase 3.2/300. Labels and normalization as in (A)-(C).

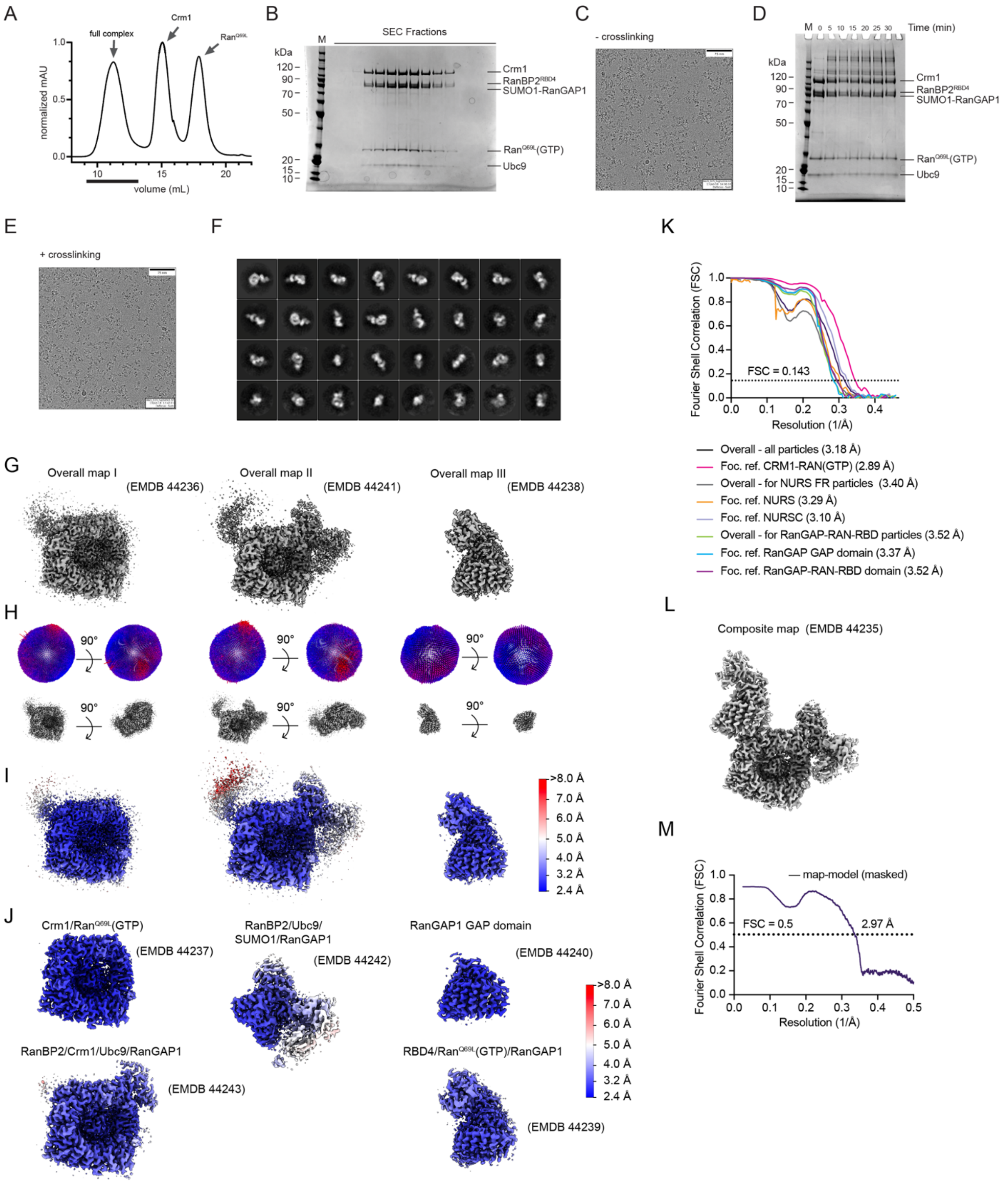

Supplementary Figure 2. Size exclusion chromatography and corresponding SDS-PAGE fractions of RanBP2^{RBD4}/SUMO1-RanGAP1/Ubc9/Crm1/Ran^{Q69L}(GTP) used for cryo-EM and quality of cryo-EM reconstructions

A Size exclusion chromatography profile of RanBP2^{RBD4}/SUMO1-RanGAP1/Ubc9/Crm1/Ran^{Q69L}(GTP) run on the Superdex Increase 10/300 GL column. Fractions corresponding to the underlined volume were run on SDS-PAGE in (B).

B Coomassie-stained SDS-PAGE gel of size exclusion fractions of RanBP2^{RBD4}/SUMO1-RanGAP1/Ubc9/Crm1/Ran^{Q69L}(GTP) that were pooled, concentrated, and used for grid preparation.

C Representative micrographs showing the appearance of particles on grids without crosslinking.

D Crosslinking time course of RanBP2^{RBD4}/SUMO1-RanGAP1/Ubc9/Crm1/Ran^{Q69L}(GTP) with 250 μM of BS3. Reaction was quenched after 30 mins before grid preparation.

E Representative micrographs showing the appearance of particles on grids after BS3 crosslinking.

F Representative 2D class averages of the RanBP2^{RBD4}/SUMO1-RanGAP1/Ubc9/Crm1/Ran^{Q69L}(GTP) complex.

G-I Overall maps (G; see Supplementary Fig. 3), their angular distribution plots (H), and their local resolution estimates (I).

J Focused refinement maps (see Supplementary Fig. 3) and their local resolution estimates.

K FSC curves calculated between half maps from the indicated reconstructions.

L Composite map of RanBP2^{RBD4}/SUMO1-RanGAP1/Ubc9/Crm1/Ran^{Q69L}(GTP).

M FSC curve calculated between the final refined model and the composite map.

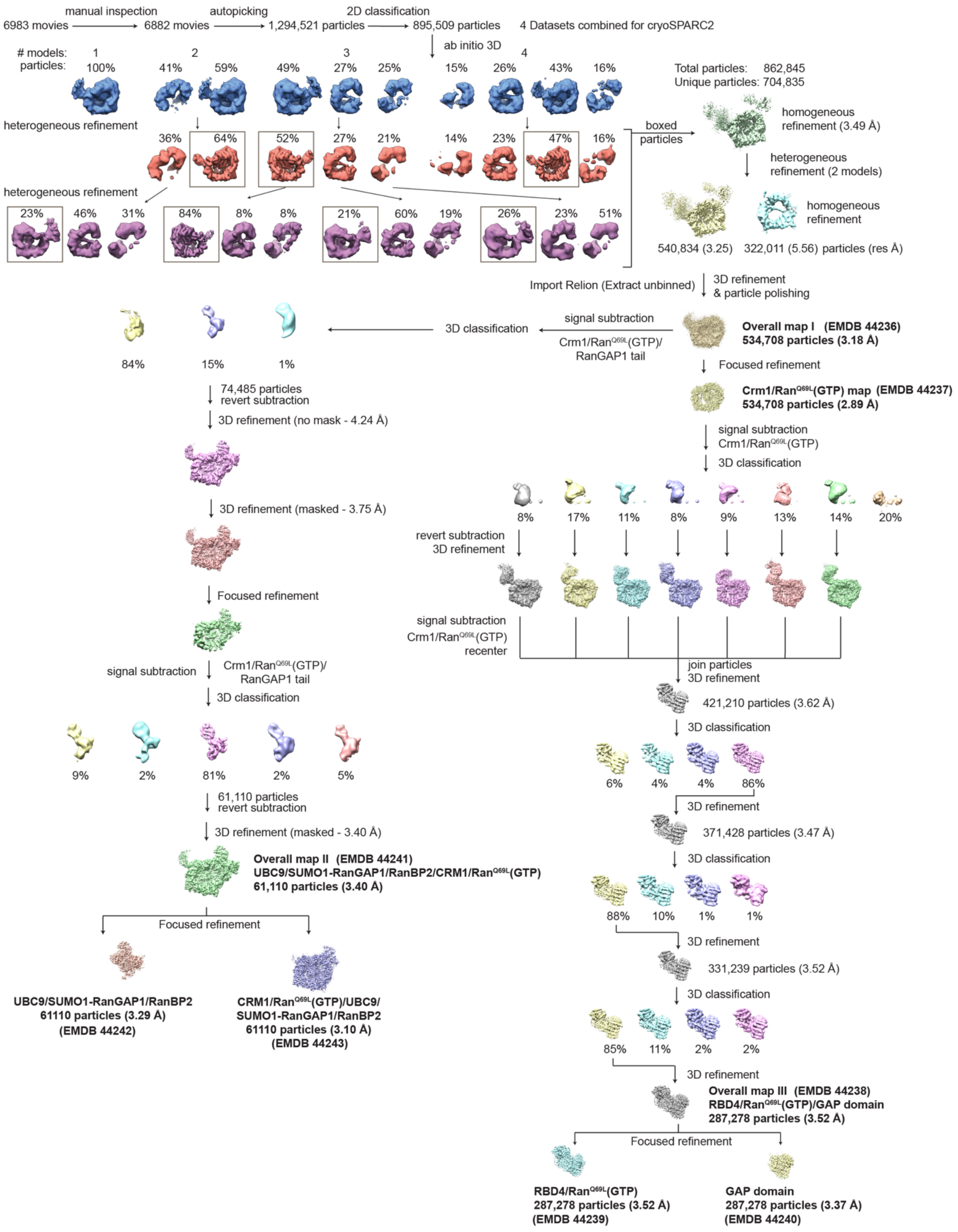

Supplementary Figure 3 – Cryo-EM data collection and processing workflow used to generate overall and composite maps

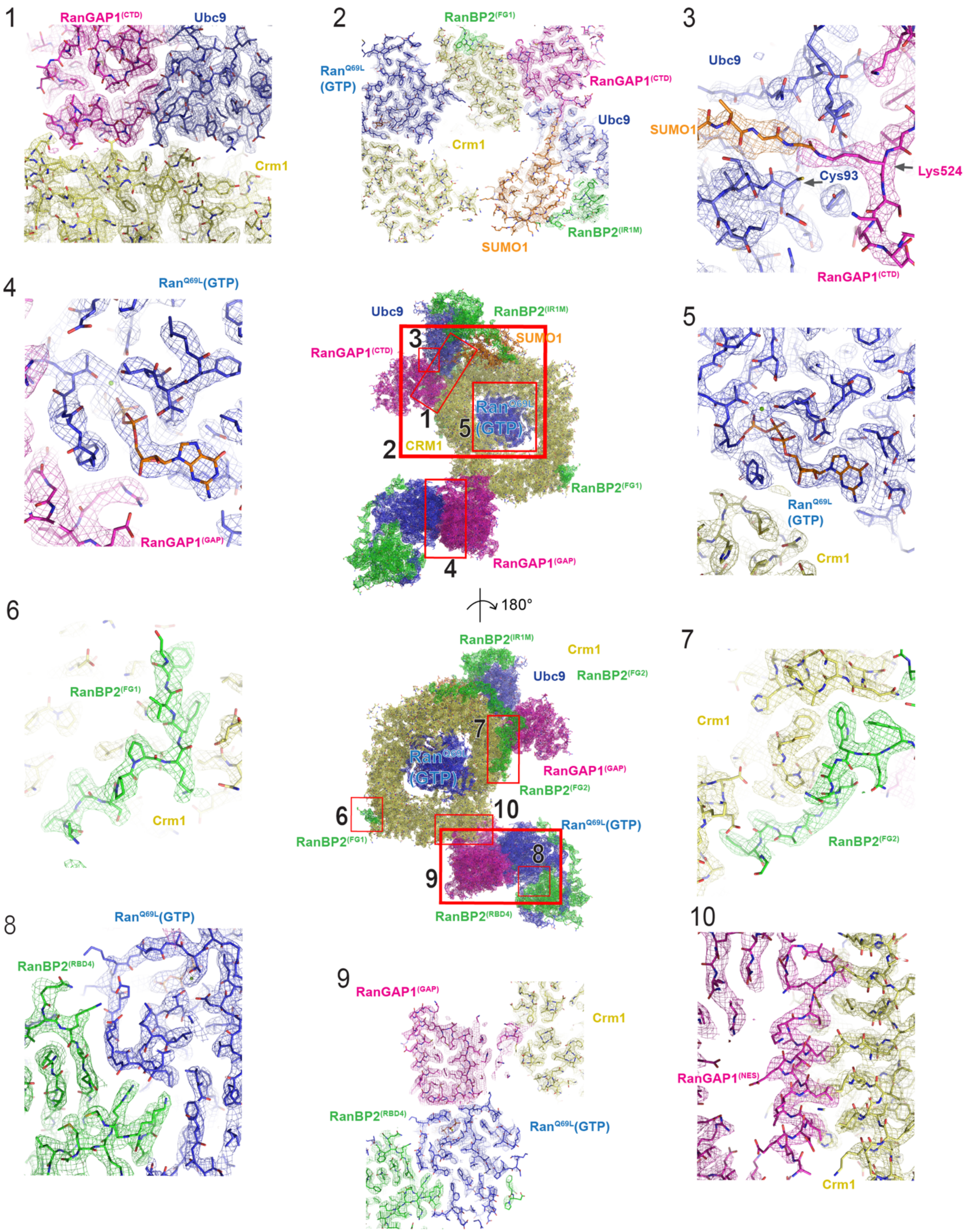

Supplementary Figure 4. Examples of EM density maps. Two overall views of the RanBP2^{RBD4}/SUMO1-RanGAP1/Ubc9/Crm1/Ran^{Q69L}(GTP) complex (middle) with boxed regions

magnified and shown in detail in the numbered panels. Ubc9 catalytic cysteine and RanGAP1 lysine conjugated to SUMO1 are noted in panel 3. Coloring same as in Fig. 2.

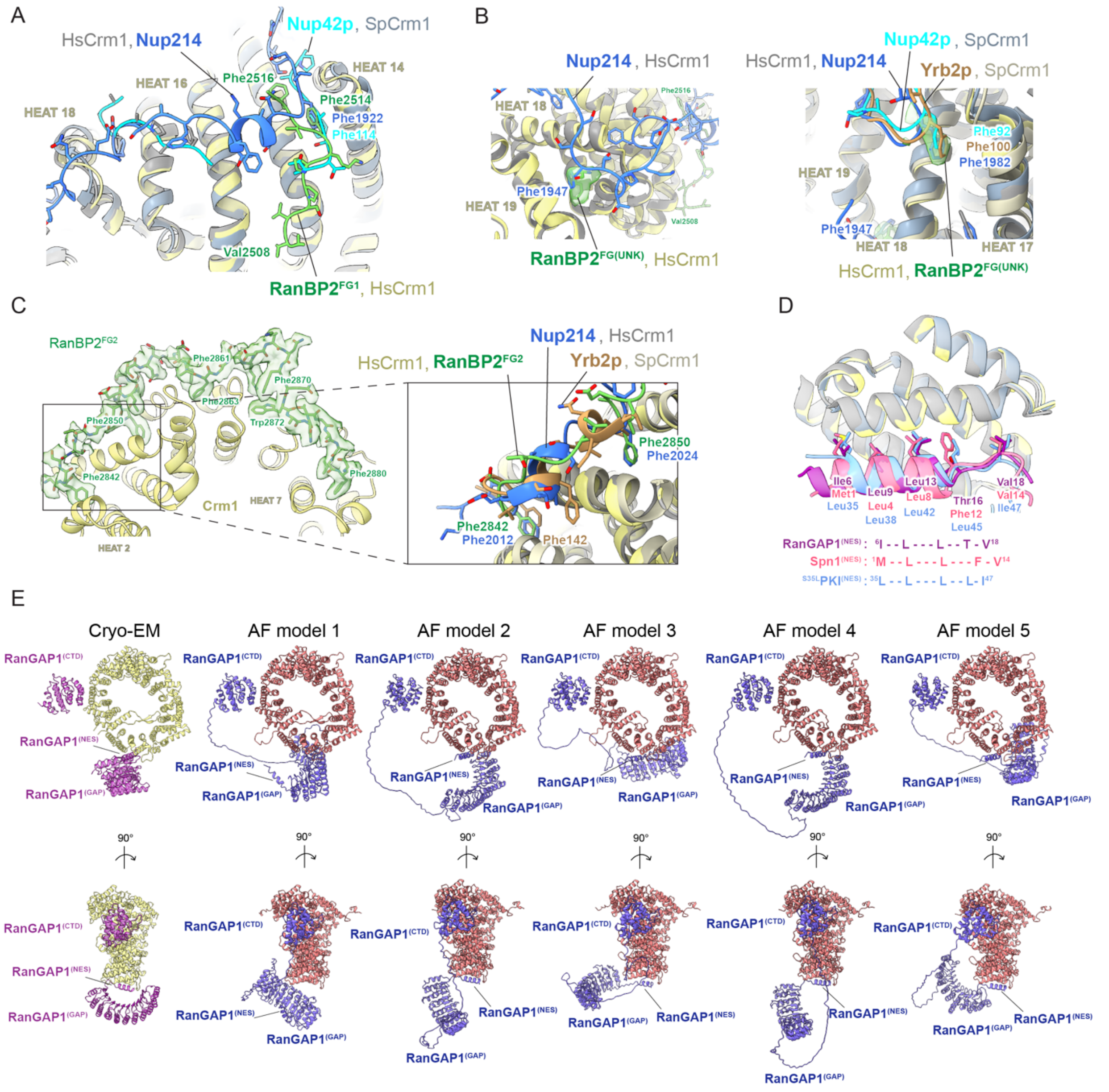

Supplementary Figure 5. Comparisons of RanBP2 FG repeats to those of other FG-repeat containing proteins and predictions of RanGAP1-Crm1 interfaces by AlphaFold multimer.

A Superpositions of the RanBP2 FG1 region with the FG repeats in previously determined structures for the human Nup214-Crm1-SPN1-Ran(GTP) (PDB 5DIS)[51] and *S. pombe* Nup42p-Crm1-PKI-Ran(GTP) (PDB 5XOJ)[53] complexes. Crm1 molecules were structurally aligned (represented in cartoon) and all chains individually colored and indicated. Residues discussed in the text are labelled.

B Isolated, unassigned densities near the RanBP2^{FG1} region (RanBP2$^{FG(UNK)}$) observed in our cryo-EM reconstruction and the corresponding phenylalanines modelled in previously determined structures. Structures colored and indicated as in A with the addition of the yeast Yrb2p-Crm1-Ran(GTP) complex (PDB 3WYF).[54] Residues discussed in the text are labelled.

C Magnified view of a region of RanBP2^{FG2} near HEAT repeats 2-4 and overlaid structures of FG repeats from Nup214 and Yrb2p as colored in B. Residues discussed in the text are labelled.

D Superposition of the NES from RanGAP1 with the NES motifs from SPN1 (PDB 5DIS)[51] and ^{S35L}PKI (PDB 5XOJ).[53] Crm1 molecules were structurally aligned (represented in cartoon) and colored as in A and B and individual NES helices were colored as indicated. Key residues comprising the NES motifs are indicated in stick representation with the sequences indicated below.

E AlphaFold2 multimer predictions for RanGAP1-Crm1 interactions. Full-length RanGAP1 and Crm1 sequences were used as inputs for AlphaFold multimer, generating 5 models, each of which contained 5 predictions. The highest confidence predictions (as judged by ipTM + pTM scores) are shown for each model. All 5 models predicted the RanGAP1^{CTD}-Crm1 interaction and 4 of the 5 predicted the RanGAP1^{NES}-Crm1 interaction (models 2-5). When taking into account all 25 predictions, 24 of 25 show the CTD interaction and 17 of 25 show the NES interaction.

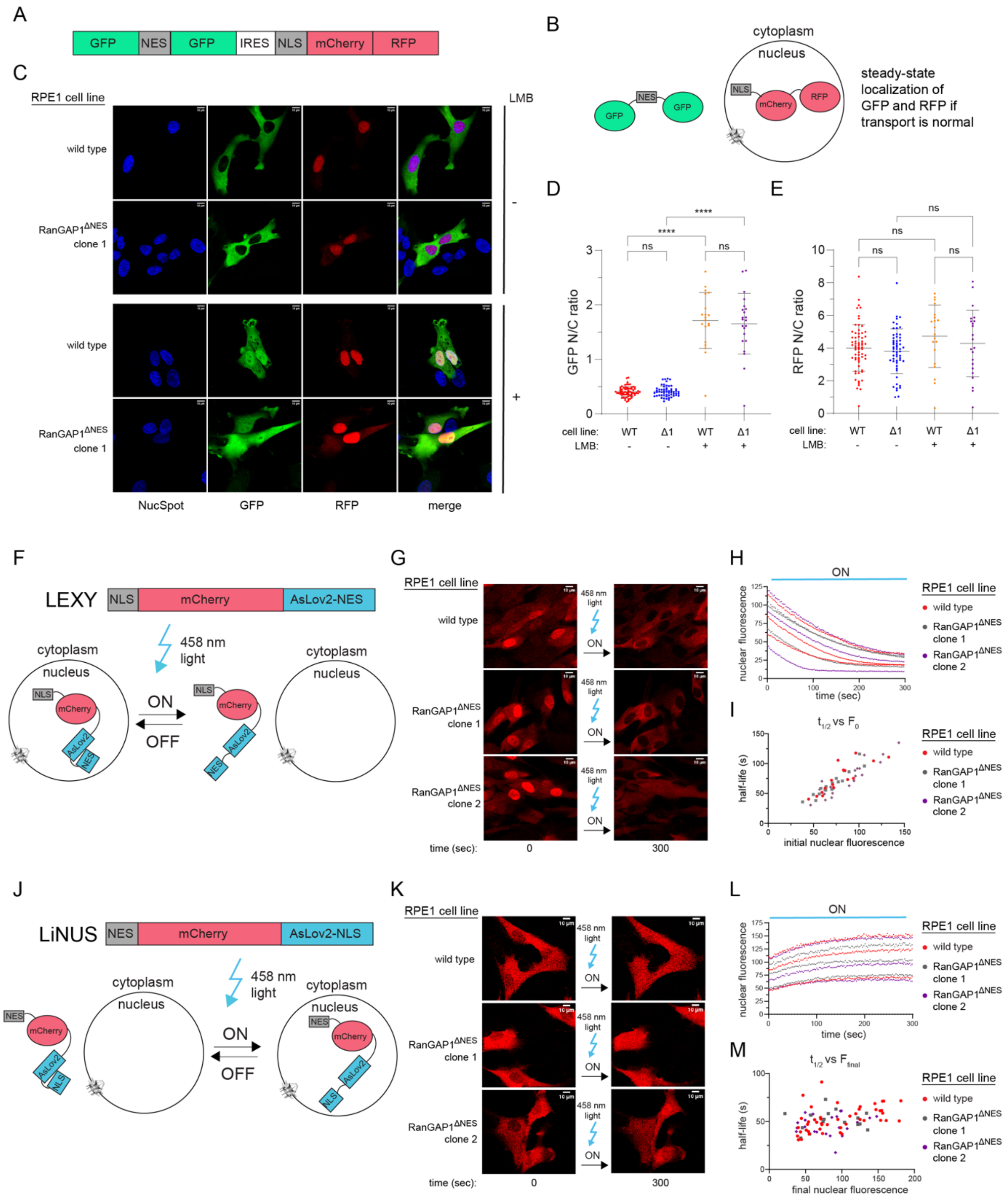

Supplementary Figure 6. Engineered cells expressing the RanGAP1$^{\Delta NES}$ variant do not exhibit overt defects in nucleocytoplasmic transport.

A Construct encoding 2xGFP-NES-IRES-2xRFP-NLS[61] used to generate stable RPE1 cell lines in cell lines expressing wild type RanGAP1 or RanGAP1$^{\Delta NES}$.

B Expected localization for GFP and RFP in cells exhibiting normal NCT.

C Representative GFP and RFP average confocal z-stack images of live wild type RPE1 and RanGAP1$^{\Delta NES}$ clone 1 cells in the absence (-) or presence (+) of 50 nM LMB. Brightness/contrast adjusted for individual channels.

D Quantification of N/C ratios for GFP for wild type and RanGAP1$^{\Delta NES}$ clone 1 cells in the absence (-) or presence (+) of 50 nM LMB. Individual points plotted and bars represent means +/- SD. Statistical significance calculated and p-values defined as in Fig. 6 (F and H). See Source Data for exact p-values. The total number of cells analyzed across at least 3 biological replicates was n = 65 (WT, no LMB), n = 56 (Δ1, no LMB), n = 20 (WT + LMB), n = 22 (Δ1 + LMB).

E Quantification of N/C ratios for RFP for wild type and RanGAP1$^{\Delta NES}$ clone 1 cells in the absence (-) or presence (+) of 50 nM LMB. Individual points plotted and bars represent means +/- SD. Same cells analyzed as in (D) with statistical analysis same as in (D).

F Schematic of construct and experimental design for the LEXY assay.[63] mCherry fused to a constitutive NLS signal and an NES bound to and masked by the AsLov2 domain localizes primarily to the nucleus in the dark (OFF). Upon illumination with 458 nm light (ON), the NES is exposed by disengaging from the AsLov2 domain resulting in the export of mCherry into the cytoplasm.

G Example images of mCherry in live wild type RPE1, RanGAP1$^{\Delta NES}$ clone 1, and RanGAP1$^{\Delta NES}$ clone 2 cell lines stably expressing the LEXY construct before exposure (time = 0) and 5 minutes after pulsatile exposure (time = 300 seconds) to 458 nm light.

H Representative data generated by quantifying nuclear mCherry fluorescence in LEXY cells at 2.57 second intervals during 458 nm light exposure (see Methods for details).

I	Plots of half-lives vs. initial nuclear fluorescence calculated from curves (fit to first order exponential models in Prism) such as shown in (H). Note that although the half-life for mCherry export increases with increasing initial mCherry nuclear fluorescence, no difference is observed for wild type and RanGAP1$^{\Delta NES}$ cells across a wide range of initial nuclear mCherry fluorescence levels.

J	Schematic of construct and experimental design for the LiNUS assay.[62] mCherry fused to a constitutive NES signal and an NLS masked by the AsLov2 domain localizes primarily to the cytoplasm in the dark (OFF). Upon illumination with 458 nm light, the NLS is exposed resulting in nuclear translocation of mCherry (ON).

K	Example images of mCherry in wild type RPE1, RanGAP1$^{\Delta NES}$ clone 1, and RanGAP1$^{\Delta NES}$ clone 2 cell lines stably expressing the LiNUS construct before exposure (time = 0) and 5 minutes after pulsatile exposure (time = 300 seconds) to 458 nm light.

L	Representative data generated by quantifying nuclear mCherry fluorescence in LiNUS cells at 2.57 second intervals during 458 nm light exposure (see Methods for details).

M	Plots of half-lives vs. final nuclear fluorescence calculated from curves (fit to first order exponential models in Prism) such as shown in (L). No difference is observed for wild type and RanGAP1$^{\Delta NES}$ cells.

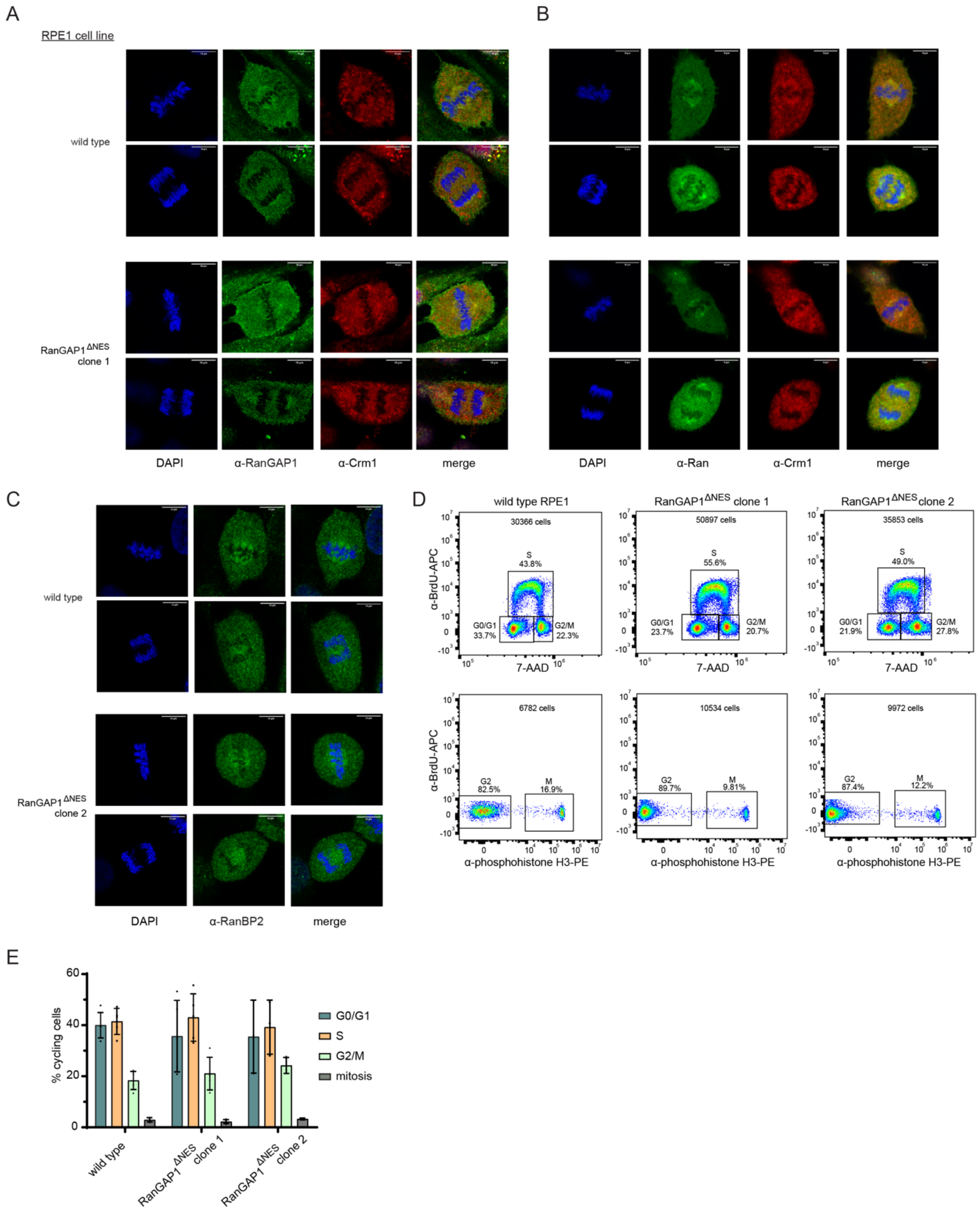

Supplementary Figure 7. **Cells expressing the RanGAP1$^{\Delta NES}$ variant exhibit the same mitotic localization of RanGAP1, Crm1, Ran, and RanBP2 as wild type cells and do not show overt cell cycle defects**.

A	Confocal microscopy images of fixed metaphase and anaphase wild type RPE1 (top) and RanGAP1$^{\Delta NES}$ clone 1 cells (bottom) co-stained with α-RanGAP1 and α-Crm1 antibodies and counterstained by DAPI. RanGAP1 detected with rabbit α-RanGAP1 polyclonal primary antibody (Bethyl, A302-027A) and alpaca α-rabbit IgG-Alexa488 secondary nanobody (Thermo, SA5-10323), both at 1:1000 dilution. Crm1 detected with mouse monoclonal α-Crm1 antibody directly labelled with Alexa647 (Santa Cruz, SC74454) used at 1:40 dilution. Representative 2D images are shown; brightness/contrast adjusted for individual channels.

B	Confocal microscopy images of fixed metaphase and anaphase wild type RPE1 (top) and RanGAP1$^{\Delta NES}$ clone 1 cells (bottom) co-stained with α-Ran and α-Crm1 antibodies and counterstained by DAPI. Ran detected with rabbit polyclonal α-Ran antibody (Proteintech, 10469-1-AP) at a 1:100 dilution and alpaca α-rabbit IgG-Alexa488 secondary nanobody (Thermo, SA5-10323) at 1:1000 dilution. Crm1 detected as in (A) but antibody used at 1:300 dilution. Representative z-stack average projections are shown; brightness/contrast adjusted for individual channels.

C	Confocal images of fixed metaphase and anaphase wild type RPE1 (top) and RanGAP1$^{\Delta NES}$ clone 2 cells stained with α-RanBP2 antibody and counterstained by DAPI. RanBP2 was detected with a monoclonal mouse α-RanBP2 antibody (Santa Cruz, sc-74518) used at 1:100 dilution and donkey anti-mouse IgG-Alexa647 secondary antibody (H+L Thermo A32787) used at 1:1000 dilution. Representative 2D images are shown; brightness/contrast adjusted for individual channels.

D	Representative FACS plots for wild type RPE1, RanGAP1$^{\Delta NES}$ clone 1, and RanGAP1$^{\Delta NES}$ clone 2 cell lines. Distribution of G0/G1, S, G2/M phases shown in BrdU vs. 7-AAD plots (top); cells in mitosis shown in BrdU vs. phosphohistone H3 plots (bottom). Boxed regions assigned to indicated cell cycle phases and cells within were quantified as indicated. Note that the indicated percentage of cells in mitosis is out of the G2/M population.

E Quantification of FACS data from plots such as those shown in C. Data from individual biological replicates ($n \geq 3$) plotted. Bars represent means +/- SD.

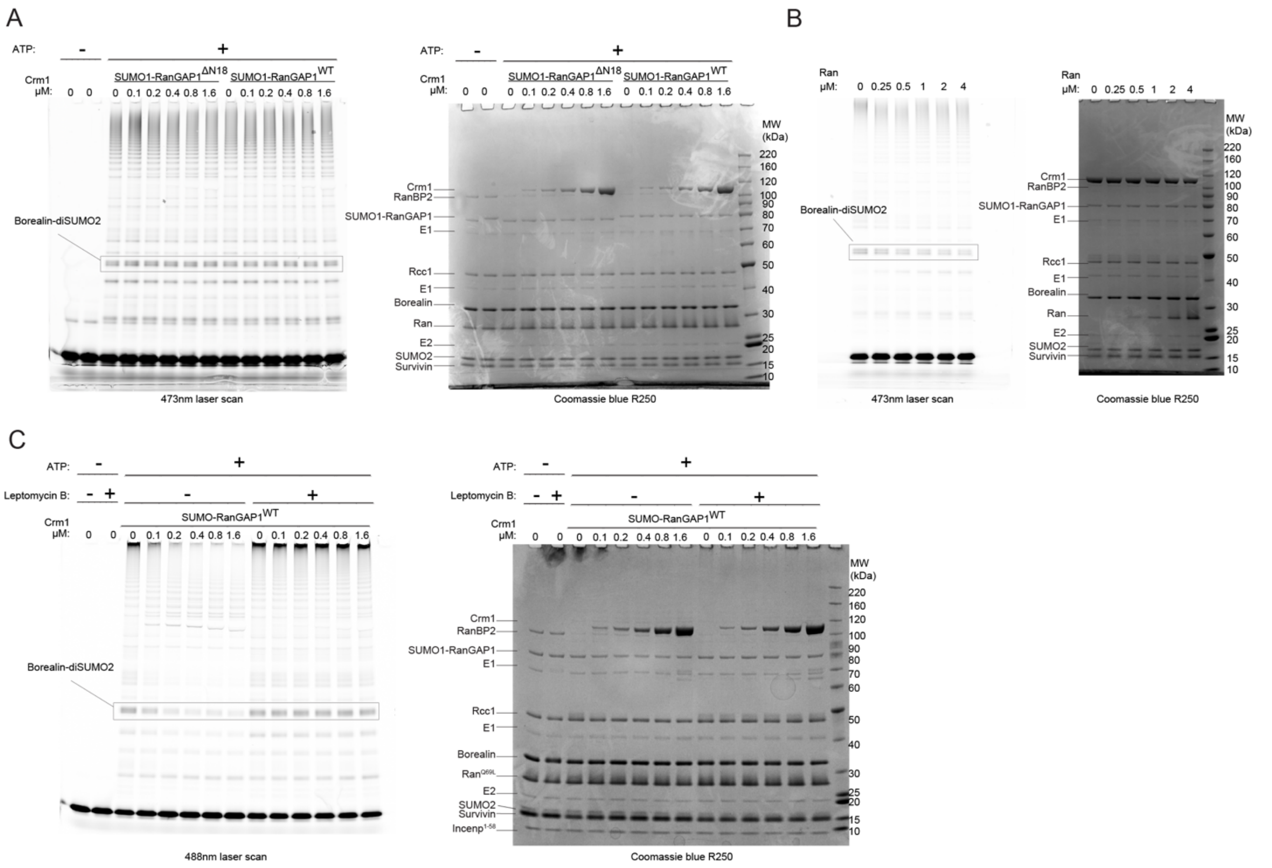

Supplementary Figure 8. **The effect of wild type Ran and leptomycin B on SUMO conjugation**

A Crm1 does not inhibit SUMO conjugation in an NES-dependent manner in the presence of wild type Ran(GTP). Crm1 was titrated into reactions containing 3.2 µM wild type Ran(GTP) and RanBP2^{RBD3-4} complexes containing either SUMO1-RanGAP1$^{\Delta N18}$ or wild type SUMO1-RanGAP1 as in Fig. 7F. Assays were done as for Ran^{Q69L}(GTP) in Fig. 7F except reactions were quenched after 50 minutes at 37 °C. Gels were scanned and stained as in Fig. 7F.

B Wild type Ran(GTP) inhibits SUMO conjugation. Ran(GTP) was titrated at the indicated concentrations into reactions containing wild type RanBP2^{RBD3-4}/SUMO1-RanGAP1/Ubc9/Crm1 complexes and reactions were done under the same conditions as in Fig. 7D. Gels were scanned and stained as in Fig. 7D.

C Leptomycin B relieves the Crm1 and NES-dependent inhibition observed in the presence of Ran^{Q69L}(GTP) in complexes with wild type SUMO1-RanGAP1. Crm1 was titrated into reactions containing 3.2 μM Ran^{Q69L}(GTP) and RanBP2^{RBD3-4}/SUMO1-RanGAP1/Ubc9 in the absence (-) or presence (+) of 2 μM leptomycin B. All reactions were done as in Fig. 7F. Gel was visualized via Alexa 488 fluorescence (left) and Coomassie Blue (right).

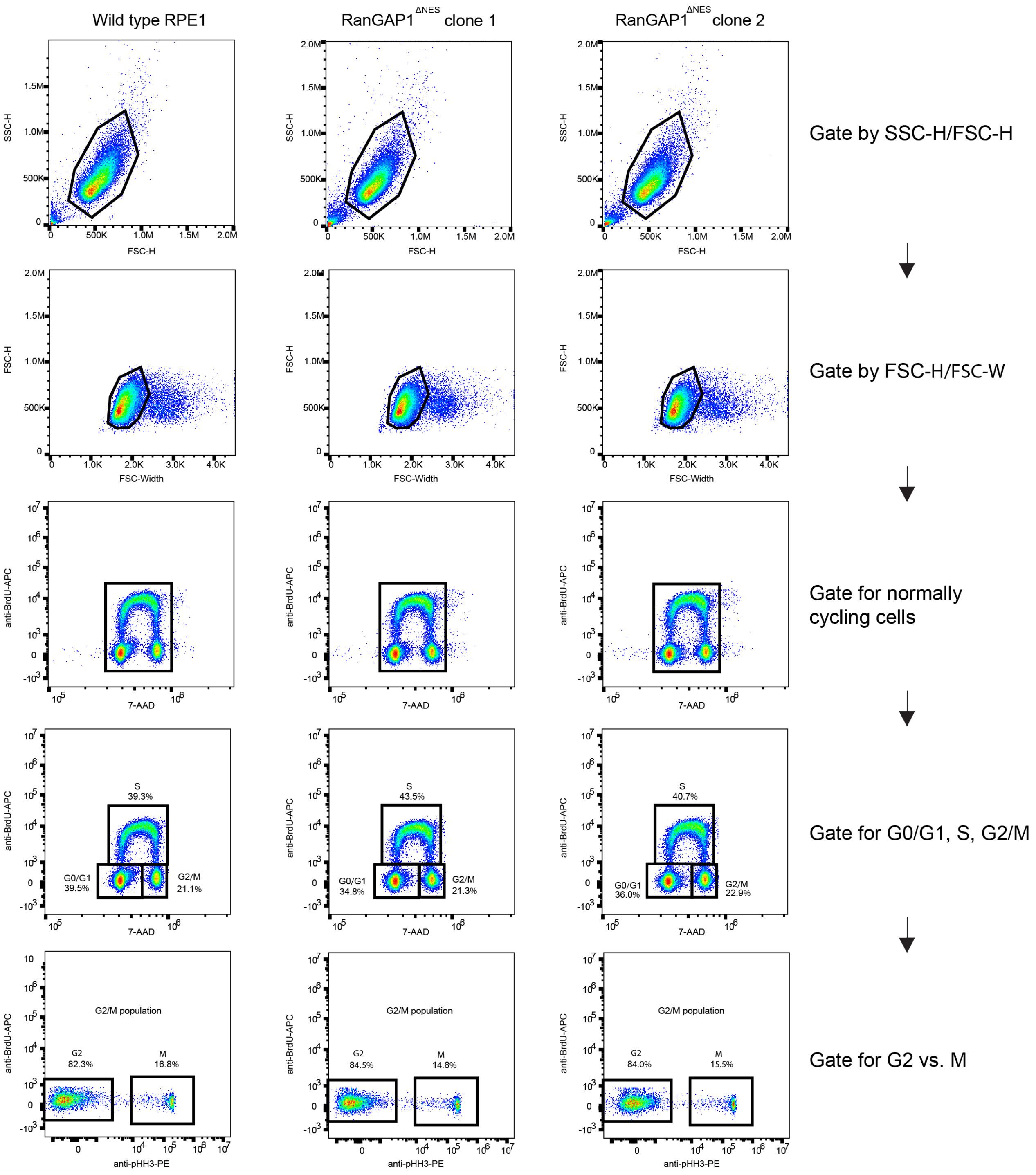

Supplementary Figure 9. Representative flow cytometry gating strategy for cell cycle analysis for wild type RPE1 and RanGAP1$^{\Delta NES}$ clones 1 and 2 (See Supplementary Fig. 7 (D-E)).

Supplementary Table 1. EM data and refinement

	RanBP2/Ubc9/SUMO1-RanGAP1/Ran(GTP)/Crm1/Ran(GTP) complex		
**Data collection**	Datasets 1 - 4		
Microscope	Titan Krios G2		
Detector/Mode	Gatan K2		
Mode	Counting - Super Resolution		
Data collection software	Serial EM		
Energy Filter	n/a		
Magnification	22,500x		
Voltage (kV)	300		
Electron exposure (e^{-}/Å^2)	85.2		
Frames	50		
Exposure Time (s)	10		
Defocus range (μm)	-1.0 to -3.0		
Super-resolution pixel size (Å)	0.544		
Fourier cropped pixel size (Å)	1.088		
Frames (collected/used)	6983/6882		
Initial particle projections (#)	540,834		
**Reconstructions**	Overall I (All components)	Overall II (All components after subclassification for Ubc9/SUMO1-RanGAP1/Ran(GTP))	Overall III (All components after subtraction for RanBP2-RBD4/Ran(GTP)/RanGAP1-GAP)
Final particle projections (#)	534,708	61,110	287,278
Symmetry	C1	C1	C1
Map resolution (Å) FSC threshold = 0.143	3.18	3.40	3.52
Map resolution range (Å) Box (contoured)	2.44-10.70 (2.59-7.39; 0.055 sig)	2.56-11.82 (2.70-8.58; 0.035 sig)	2.57-11.86 (2.74-6.04; 0.045 sig)
Map sharpening B factor (Å^2)	-82.11	-55.18	-135.37
Sphericity (FSC threshold = 0.5)	0.975	0.764	0.980
EMDB	44236	44241	44238
**Focused Refinement Reconstructions**	Crm1/Ran(GTP)/RanBP2-FG/RanGAP1-NES	RanBP2-IR/Ubc9/SUMO1- RanGAP1-CTD	RanBP2-RBD4/Ran(GTP)
Final particle projections (#)	534,708	61,110	287,278
Symmetry	C1	C1	C1
Map resolution (Å) FSC threshold = 0.143	2.89	3.29	3.52
Map resolution range (Å) Box (contoured)	2.32-9.80 (2.42-5.59; 0.050 sig)	2.96-11.11 (2.95-6.05; 0.025 sig)	2.67-11.33 (2.86-6.20; 0.045 sig)
Map sharpening B factor (Å^2)	-63.36	-86.25	-142.82
Sphericity (FSC threshold = 0.5)	0.980	0.920	0.976
EMDB	44237	44242	44239
**Focused Refinement Reconstructions**		RanBP2/Crm1/Ran(GTP)/Ubc9/SUMO1-RanGAP1-CTD	RanGAP1-GAP
Final particle projections (#)		61,110	287,278
Symmetry		C1	C1
Map resolution (Å) FSC threshold = 0.143		3.10	3.37
Map resolution range (Å) Box (contoured)		2.50-10.48 (2.69-6.52; 0.045 sig)	2.63-11.98 (2.63-4.02; 0.045 sig)
Map sharpening B factor (Å^2)		-53.40	-154.44
Sphericity (FSC threshold = 0.5)		0.968	0.974
EMDB		44243	44240
**Refinement**			
Initial models used (PDB code)	3UIN, 3GJX, 4L6E, 1K5D		
Model resolution (Å) FSC threshold = 0.5	2.97		
EMDB (Composite map)	44235		
Final model (PDB code)	9B62		
**Model composition**			
Non-hydrogen atoms	19,287		
Protein residues	2,414		
Nucleic acid residues	2		
Ligand (Mg^{2+})	2		
**Mean *B* factors**			
Protein	62.9		
Nucleic acid	40.8		
Ligand (Mg^{2+})	43.4		
**RMS deviations**			
Bond lengths (Å)	0.003		
Bond angles (º)	0.447		
**Validation**			
Molprobity score	1.43		
Clashscore	4.14		
CC volume/mask	0.82/0.84		
EMRinger score	3.78		
Rotamer Outliers (%)	1.54		
C-beta deviations (%)	0		
CaBLAM outliers (%)	1.18		
**Ramachandran plot**			
% favored	92.11		
% allowed	7.89		
% outliers	0		
