# Supplementary material for: Structural basis for a nucleoporin exportin complex between RanBP2, SUMO1-RanGAP1, the E2 Ubc9, Crm1 and the Ran GTPase
Source: Nat Commun. 2025 Jul 11;16:6403. doi: 10.1038/s41467-025-61694-1 (PMC12246119; doi:10.1038/s41467-025-61694-1)
Supplement: Supplementary file 3 — Reporting Summary [file 41467_2025_61694_MOESM3_ESM.pdf]

nature portfolio

Corresponding author(s): Christopher D. Lima

Last updated by author(s): Jun 16, 2025

Reporting Summary

Nature Portfolio wishes to improve the reproducibility of the work that we publish. This form provides structure for consistency and transparency in reporting. For further information on Nature Portfolio policies, see our Editorial Policies and the Editorial Policy Checklist.

Statistics

For all statistical analyses, confirm that the following items are present in the figure legend, table legend, main text, or Methods section.

n/a	Confirmed	
☐	☒	The exact sample size (*n*) for each experimental group/condition, given as a discrete number and unit of measurement
☐	☒	A statement on whether measurements were taken from distinct samples or whether the same sample was measured repeatedly
☐	☒	The statistical test(s) used AND whether they are one- or two-sided<br>*Only common tests should be described solely by name; describe more complex techniques in the Methods section.*
☒	☐	A description of all covariates tested
☐	☒	A description of any assumptions or corrections, such as tests of normality and adjustment for multiple comparisons
☐	☒	A full description of the statistical parameters including central tendency (e.g. means) or other basic estimates (e.g. regression coefficient) AND variation (e.g. standard deviation) or associated estimates of uncertainty (e.g. confidence intervals)
☐	☒	For null hypothesis testing, the test statistic (e.g. *F*, *t*, *r*) with confidence intervals, effect sizes, degrees of freedom and *P* value noted<br>*Give P values as exact values whenever suitable.*
☒	☐	For Bayesian analysis, information on the choice of priors and Markov chain Monte Carlo settings
☒	☐	For hierarchical and complex designs, identification of the appropriate level for tests and full reporting of outcomes
☒	☐	Estimates of effect sizes (e.g. Cohen's *d*, Pearson's *r*), indicating how they were calculated

Our web collection on statistics for biologists contains articles on many of the points above.

Software and code

Policy information about availability of computer code

Data collection	Cryo-EM data was collected with SerialEM software. Confocal microscopy images were collected with LASX software. Flow cytometry data was collected with CytExpert software.
Data analysis	Cryo-EM data was processed and analyzed with MotionCor2, Gctf, Relion 3.0 and 3.1.2, cryoSPARC2, Phenix suite, Coot, Molprobity, ChimeraX 1.8, and Pymol 2.5.4. Flow cytometry data was analyzed in FlowJo 10. Microscopy images were processed in Fiji 1.53t and quantification performed in CellProfiler 4.2.5. Sequence alignment was done in Jalview 2.11.3.3 via the T-coffee alignment module using default settings. Statistical analysis was done in Prism 10.

For manuscripts utilizing custom algorithms or software that are central to the research but not yet described in published literature, software must be made available to editors and reviewers. We strongly encourage code deposition in a community repository (e.g. GitHub). See the Nature Portfolio guidelines for submitting code & software for further information.

Data

Policy information about availability of data

All manuscripts must include a data availability statement. This statement should provide the following information, where applicable:

- Accession codes, unique identifiers, or web links for publicly available datasets
- A description of any restrictions on data availability
- For clinical datasets or third party data, please ensure that the statement adheres to our policy

All maps and coordinates have been deposited and are available at the EMDB (ebi.ac.uk/emdb/) and PDB (rcsb.org) data banks via the following accession codes: PDB:9B62; EMDB-44235; EMDB-44236; EDMB-44237; EMDB-44238; EMDB-44239; EMDB-44240; EMDB-44241; EMDB-44242; EMDB-44243

Research involving human participants, their data, or biological material

Policy information about studies with human participants or human data. See also policy information about sex, gender (identity/presentation), and sexual orientation and race, ethnicity and racism.

Reporting on sex and gender	N/A
Reporting on race, ethnicity, or other socially relevant groupings	N/A
Population characteristics	N/A
Recruitment	N/A
Ethics oversight	N/A

Note that full information on the approval of the study protocol must also be provided in the manuscript.

Field-specific reporting

Please select the one below that is the best fit for your research. If you are not sure, read the appropriate sections before making your selection.

☒ Life sciences ☐ Behavioural & social sciences ☐ Ecological, evolutionary & environmental sciences

For a reference copy of the document with all sections, see nature.com/documents/nr-reporting-summary-flat.pdf

Life sciences study design

All studies must disclose on these points even when the disclosure is negative.

Sample size	Sample sizes were not predetermined. Cryo-EM sample size was determined by available microscope time. Number of cells analyzed by confocal microscopy was determined by available microscope time and tests for statistical significance. Flow cytometry replicate size was chosen to account for variability between experiments and ensure overall reproducibility. SUMO modification assays were done at least 3 times to ensure reproducibility.
Data exclusions	Cryo-EM movies were manually inspected and low quality movies excluded from analysis. Cryo-EM particle class averages from 2D classification that were deemed noise were excluded from analysis.
Replication	Cryo-EM data was collected from 4 grids. Quantification of nuclear to cytoplasmic ratios via confocal microscopy was performed on at least 3 independent biological replicates. Flow cytometry was done with at least 3 biological replicates and at least 10,000 cells per experiment. SUMO modification assays were done at least 3 times. Western Blots were replicated with at least 3 biological replicates.
Randomization	During Cryo-EM 3D refinements, particles were randomly split into two groups and independently refined. Gold-standard Fourier Shell Correlation of the two reconstructions was then calculated to determine resolution. Randomization was not applicable to other experiments.
Blinding	Blinding was not done when selecting cells to analyze protein localization in confocal studies since the person collecting the data also analyzed it. However, this was partially compensated by using an unbiased, semi-automated CellProfiler pipeline for image analysis (see Methods for details). Blinding was not applicable to other experiments.

Reporting for specific materials, systems and methods

We require information from authors about some types of materials, experimental systems and methods used in many studies. Here, indicate whether each material, system or method listed is relevant to your study. If you are not sure if a list item applies to your research, read the appropriate section before selecting a response.

Materials & experimental systems

n/a	Involved in the study
☐	☒ Antibodies
☐	☒ Eukaryotic cell lines
☒	☐ Palaeontology and archaeology
☒	☐ Animals and other organisms
☒	☐ Clinical data
☒	☐ Dual use research of concern
☒	☐ Plants

Methods

n/a	Involved in the study
☒	☐ ChIP-seq
☐	☒ Flow cytometry
☒	☐ MRI-based neuroimaging

Antibodies

Antibodies used

anti-RanGAP1 (Bethyl, cat# A302-027A); anti-RanGAP1 (Sigma/Prestige, cat# HPA062034); anti-Ran (BD, cat# 610340); anti-Ran (Proteintech, cat# 10469-1-AP); anti-Beta-actin (Novus, cat# NBP1-47423); anti-Beta-actin (Santa Cruz, cat# SC81178); anti-Crm1 labelled with Alexa647 (Santa Cruz, cat# SC74454); anti-rabbit IgG conjugated to HRP (Promega, cat# W401B); anti-mouse IgG conjugated to HRP (Promega, cat# W402B); anti-rabbit IgG nanobody (VHH) conjugated to Alexa488 (Thermo Fisher, cat# SA5-10323); anti-rabbit IgG nanobody (VHH) conjugated to Alexa647 (Thermo Fisher, cat# SA5-10327); anti-mouse IgG conjugated to Alexa647 (Thermo Fisher, cat# A32787); anti-histone H3 phospho (Ser10) conjugated to PE (BioLegend, cat# 650808), anti-RanBP2 (Santa Cruz, cat# SC74518)

Validation

1) Bethyl, cat# A302-027A - validation performed by Fortis - see https://www.fortislife.com/products/primary-antibodies/rabbit-anti-rangap1-antibody/BETHYL-A302-027
2) Sigma/Prestige, cat# HPA062034 - enhanced validation by Human Protein Atlas project - see https://www.sigmaaldrich.com/US/en/product/sigma/hpa062034
3) BD, cat# 610340 - cited in 65 publications - see https://www.citeab.com/antibodies/3289029-610340-bd-transduction-laboratories-purified-mouse
4) Proteintech, cat# 10469-1-AP - validated in several publications through knockdown -see https://www.ptglab.com/products/RAN-Antibody-10469-1-AP.htm
5) Novus, cat# NBP1-47423 - cited in 13 publications - see https://www.citeab.com/antibodies/466836-nbp1-47423-beta-actin-antibody-8h10d10-bsa-free?des=29f84a278f977e3a
6) Santa Cruz, cat# SC81178 - cited in over 13,000 publications - see https://www.citeab.com/antibodies/788577-sc-47778-beta-actin-antibody-c4
7) Santa Cruz, cat# SC74454 - cited in over 40 publications - see https://www.citeab.com/antibodies/784148-sc-74454-crm1-antibody-c-1
8) BioLegend, cat# 650808 - cited in 4 publications - see https://www.citeab.com/antibodies/2861729-650807-pe-anti-histone-h3-phospho-ser10-antibody?des=18b02080bd88363a
9) Santa Cruz, cat# SC74518 - cited in 33 publications - see https://www.scbt.com/p/ran-bp-2-antibody-d-4?srsltid=AfmBOoo7a6q4HP-UrL8cJhaLeSc7FGMYGkPqXqFF72MHZ2BXKyjjruAh

Eukaryotic cell lines

Policy information about cell lines and Sex and Gender in Research

Cell line source(s)	human hTERT-RPE1 - from ATCC, Cat# CRL4000
Authentication	Done by ATCC
Mycoplasma contamination	Negative for mycoplasma, tested on a regular basis
Commonly misidentified lines (See ICLAC register)	No commonly misidentified lines used

Plants

Seed stocks	N/A
Novel plant genotypes	N/A
Authentication	N/A

Flow Cytometry

Plots

Confirm that:

☒ The axis labels state the marker and fluorochrome used (e.g. CD4-FITC).

☒ The axis scales are clearly visible. Include numbers along axes only for bottom left plot of group (a 'group' is an analysis of identical markers).

☒ All plots are contour plots with outliers or pseudocolor plots.

☒ A numerical value for number of cells or percentage (with statistics) is provided.

Methodology

Sample preparation	hTERT-RPE1 cells were fixed with PFA and permeabilized with methanol. See Methods for detailed sample preparation.
Instrument	Beckman Coulter CytoFLEX LX
Software	CytExpert Software was used to collect data and FlowJo was used to analyze the data.
Cell population abundance	N/A
Gating strategy	See Supplementary Information Fig. 9

☒ Tick this box to confirm that a figure exemplifying the gating strategy is provided in the Supplementary Information.
